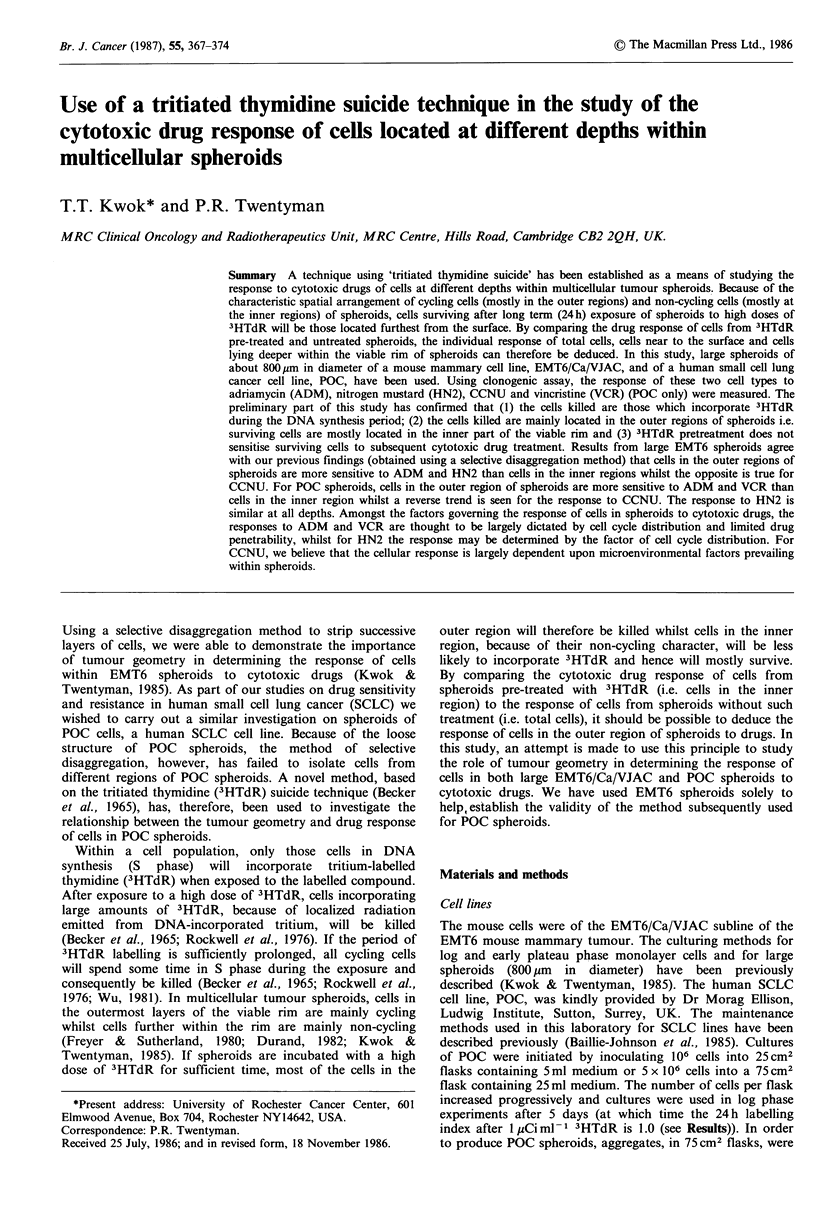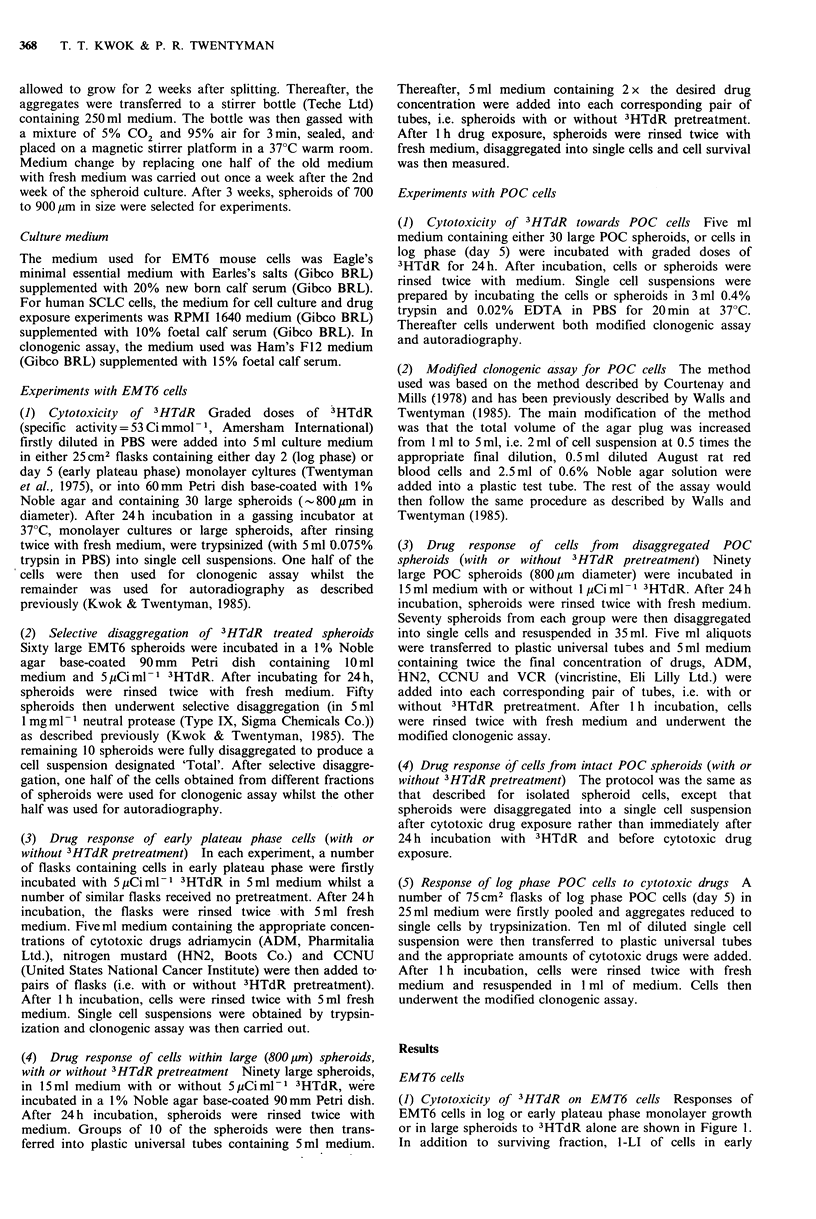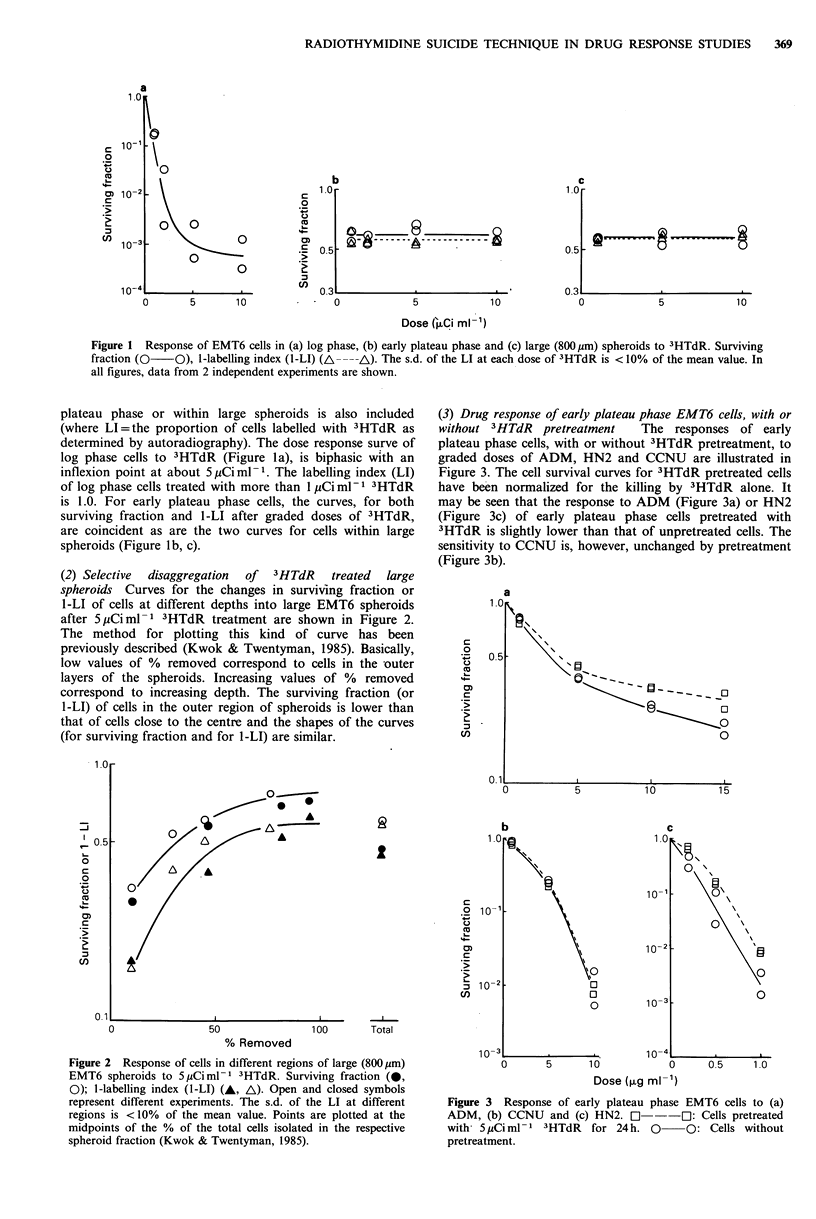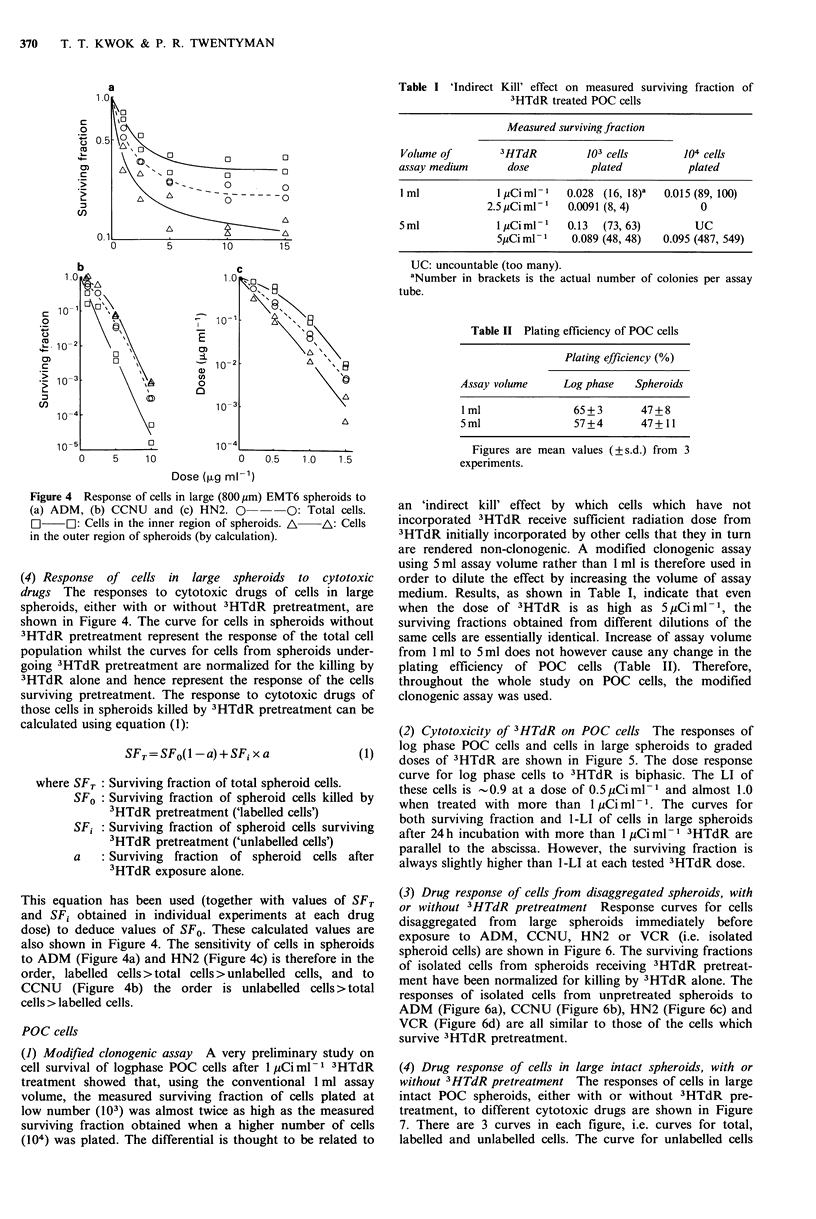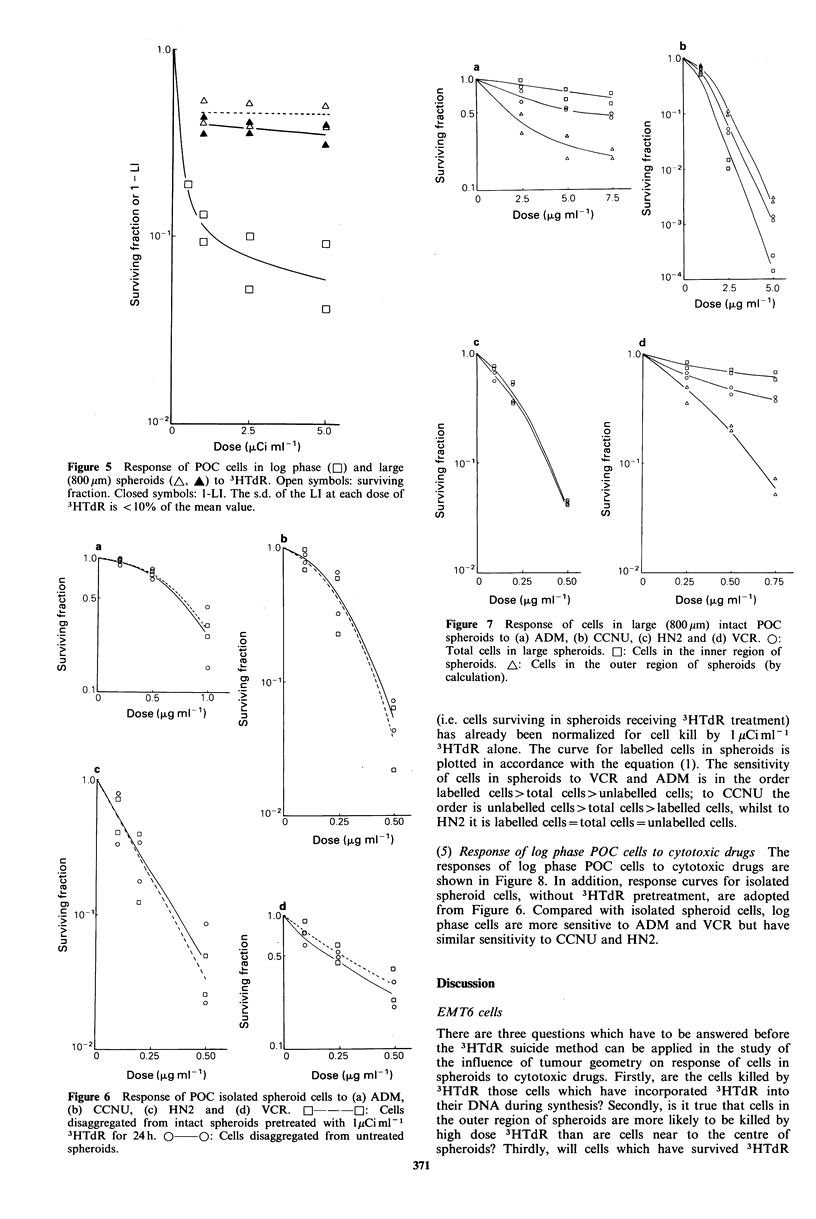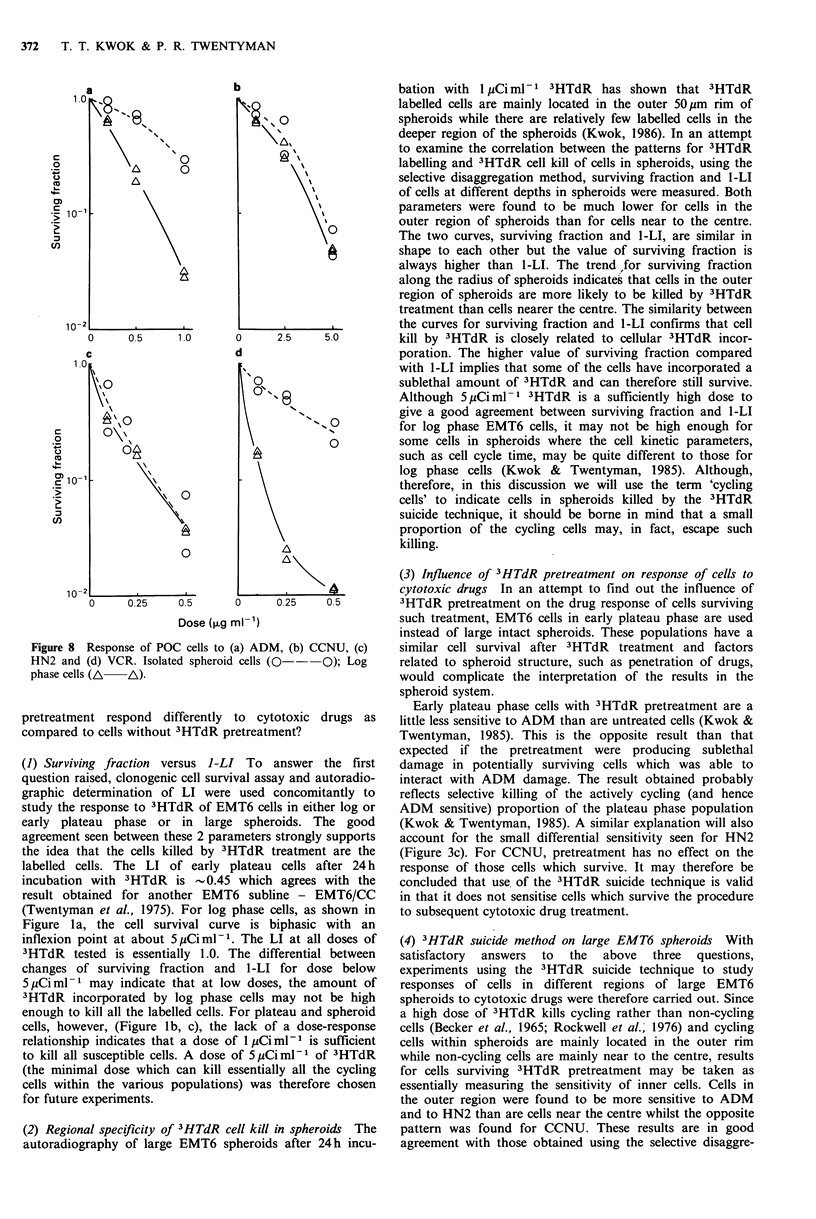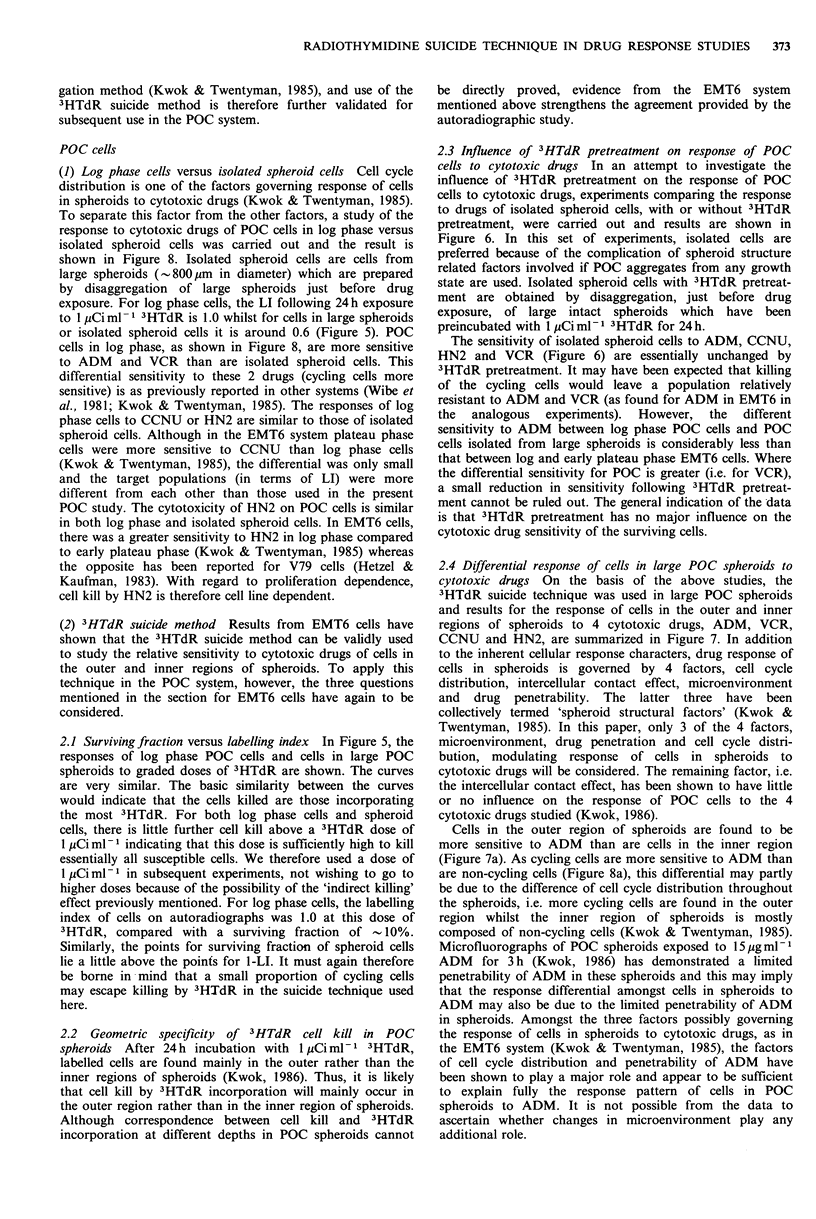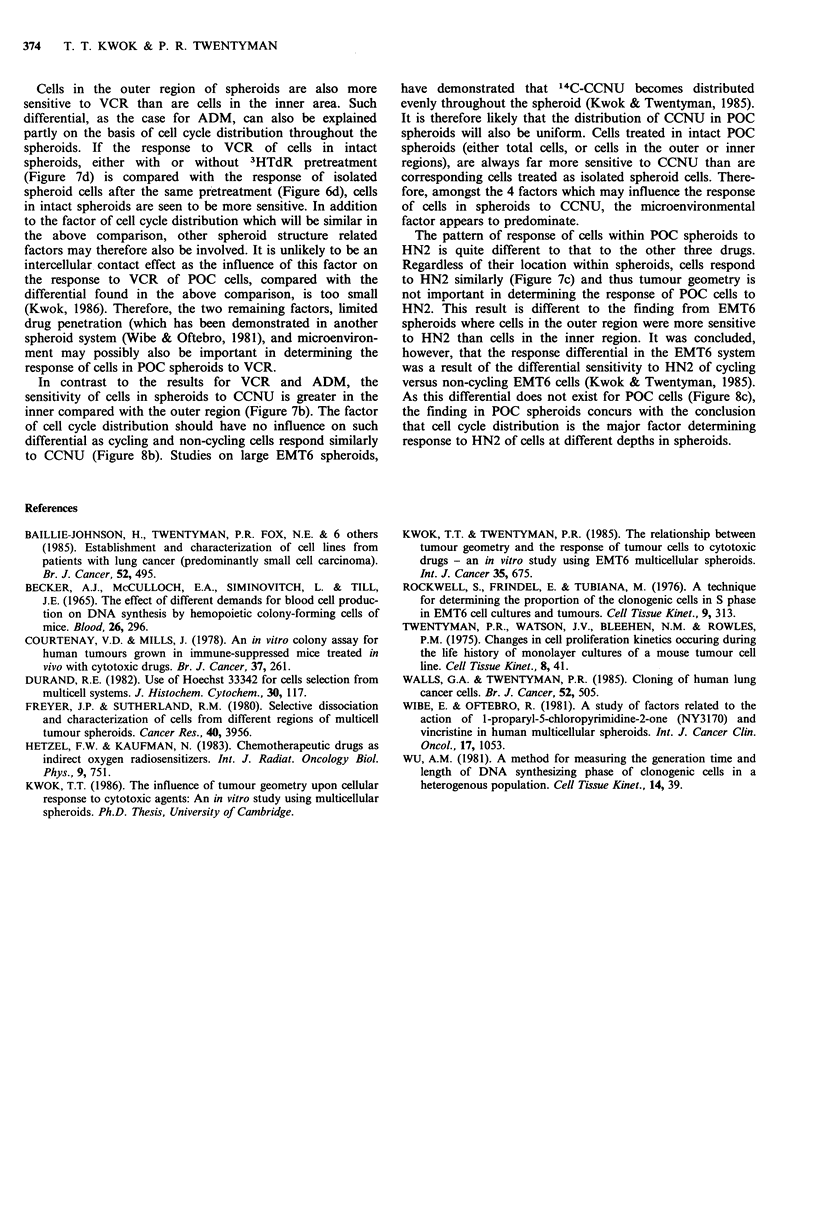# Use of a tritiated thymidine suicide technique in the study of the cytotoxic drug response of cells located at different depths within multicellular spheroids.

**DOI:** 10.1038/bjc.1987.74

**Published:** 1987-04

**Authors:** T. T. Kwok, P. R. Twentyman

## Abstract

A technique using 'tritiated thymidine suicide' has been established as a means of studying the response to cytotoxic drugs of cells at different depths within multicellular tumour spheroids. Because of the characteristic spatial arrangement of cycling cells (mostly in the outer regions) and non-cycling cells (mostly at the inner regions) of spheroids, cells surviving after long term (24 h) exposure of spheroids to high doses of 3HTdR will be those located furthest from the surface. By comparing the drug response of cells from 3HTdR pre-treated and untreated spheroids, the individual response of total cells, cells near to the surface and cells lying deeper within the viable rim of spheroids can therefore be deduced. In this study, large spheroids of about 800 micron in diameter of a mouse mammary cell line, EMT6/Ca/VJAC, and of a human small cell lung cancer cell line, POC, have been used. Using clonogenic assay, the response of these two cell types to adriamycin (ADM), nitrogen mustard (HN2), CCNU and vincristine (VCR) (POC only) were measured. The preliminary part of this study has confirmed that the cells killed are those which incorporate 3HTdR during the DNA synthesis period; the cells killed are mainly located in the outer regions of spheroids i.e. surviving cells are mostly located in the inner part of the viable rim and 3HTdR pretreatment does not sensitise surviving cells to subsequent cytotoxic drug treatment. Results from large EMT6 spheroids agree with our previous findings (obtained using a selective disaggregation method) that cells in the outer regions of spheroids are more sensitive to ADM and HN2 than cells in the inner regions whilst the opposite is true for CCNU. For POC spheroids, cells in the outer region of spheroids are more sensitive to ADM and VCR than cells in the inner region whilst a reverse trend is seen for the response to CCNU. The response to HN2 is similar at all depths. Amongst the factors governing the response of cells in spheroids to cytotoxic drugs, the responses to ADM and VCR are thought to be largely dictated by cell cycle distribution and limited drug penetrability, whilst for HN2 the response may be determined by the factor of cell cycle distribution. For CCNU, we believe that the cellular response is largely dependent upon microenvironmental factors prevailing within spheroids.


Br. J. Cancer (1987), 55, 367–374
© The Macmillan Press Ltd., 1986

### Use of a tritiated thymidine suicide technique in the study of the cytotoxic drug response of cells located at different depths within multicellular spheroids

T.T. Kwok* and P.R. Twentyman

*MRC Clinical Oncology and Radiotherapeutics Unit, MRC Centre, Hills Road, Cambridge CB2 2QH, UK.*

**Summary** A technique using 'tritiated thymidine suicide' has been established as a means of studying the response to cytotoxic drugs of cells at different depths within multicellular tumour spheroids. Because of the characteristic spatial arrangement of cycling cells (mostly in the outer regions) and non-cycling cells (mostly at the inner regions) of spheroids, cells surviving after long term (24 h) exposure of spheroids to high doses of $^3$HTdR will be those located furthest from the surface. By comparing the drug response of cells from $^3$HTdR pre-treated and untreated spheroids, the individual response of total cells, cells near to the surface and cells lying deeper within the viable rim of spheroids can therefore be deduced. In this study, large spheroids of about 800 $\mu$m in diameter of a mouse mammary cell line, EMT6/Ca/VJAC, and of a human small cell lung cancer cell line, POC, have been used. Using clonogenic assay, the response of these two cell types to adriamycin (ADM), nitrogen mustard (HN2), CCNU and vincristine (VCR) (POC only) were measured. The preliminary part of this study has confirmed that (1) the cells killed are those which incorporate $^3$HTdR during the DNA synthesis period; (2) the cells killed are mainly located in the outer regions of spheroids i.e. surviving cells are mostly located in the inner part of the viable rim and (3) $^3$HTdR pretreatment does not sensitise surviving cells to subsequent cytotoxic drug treatment. Results from large EMT6 spheroids agree with our previous findings (obtained using a selective disaggregation method) that cells in the outer regions of spheroids are more sensitive to ADM and HN2 than cells in the inner regions whilst the opposite is true for CCNU. For POC spheroids, cells in the outer region of spheroids are more sensitive to ADM and VCR than cells in the inner region whilst a reverse trend is seen for the response to CCNU. The response to HN2 is similar at all depths. Amongst the factors governing the response of cells in spheroids to cytotoxic drugs, the responses to ADM and VCR are thought to be largely dictated by cell cycle distribution and limited drug penetrability, whilst for HN2 the response may be determined by the factor of cell cycle distribution. For CCNU, we believe that the cellular response is largely dependent upon microenvironmental factors prevailing within spheroids.

Using a selective disaggregation method to strip successive layers of cells, we were able to demonstrate the importance of tumour geometry in determining the response of cells within EMT6 spheroids to cytotoxic drugs (Kwok & Twentyman, 1985). As part of our studies on drug sensitivity and resistance in human small cell lung cancer (SCLC) we wished to carry out a similar investigation on spheroids of POC cells, a human SCLC cell line. Because of the loose structure of POC spheroids, the method of selective disaggregation, however, has failed to isolate cells from different regions of POC spheroids. A novel method, based on the tritiated thymidine ($^3$HTdR) suicide technique (Becker *et al.*, 1965), has, therefore, been used to investigate the relationship between the tumour geometry and drug response of cells in POC spheroids.

Within a cell population, only those cells in DNA synthesis (S phase) will incorporate tritium-labelled thymidine ($^3$HTdR) when exposed to the labelled compound. After exposure to a high dose of $^3$HTdR, cells incorporating large amounts of $^3$HTdR, because of localized radiation emitted from DNA-incorporated tritium, will be killed (Becker *et al.*, 1965; Rockwell *et al.*, 1976). If the period of $^3$HTdR labelling is sufficiently prolonged, all cycling cells will spend some time in S phase during the exposure and consequently be killed (Becker *et al.*, 1965; Rockwell *et al.*, 1976; Wu, 1981). In multicellular tumour spheroids, cells in the outermost layers of the viable rim are mainly cycling whilst cells further within the rim are mainly non-cycling (Freyer & Sutherland, 1980; Durand, 1982; Kwok & Twentyman, 1985). If spheroids are incubated with a high dose of $^3$HTdR for sufficient time, most of the cells in the outer region will therefore be killed whilst cells in the inner region, because of their non-cycling character, will be less likely to incorporate $^3$HTdR and hence will mostly survive. By comparing the cytotoxic drug response of cells from spheroids pre-treated with $^3$HTdR (i.e. cells in the inner region) to the response of cells from spheroids without such treatment (i.e. total cells), it should be possible to deduce the response of cells in the outer region of spheroids to drugs. In this study, an attempt is made to use this principle to study the role of tumour geometry in determining the response of cells in both large EMT6/Ca/VJAC and POC spheroids to cytotoxic drugs. We have used EMT6 spheroids solely to help establish the validity of the method subsequently used for POC spheroids.

*Present address: University of Rochester Cancer Center, 601 Elmwood Avenue, Box 704, Rochester NY14642, USA.
Correspondence: P.R. Twentyman.
Received 25 July, 1986; and in revised form, 18 November 1986.

### Materials and methods

### *Cell lines*

The mouse cells were of the EMT6/Ca/VJAC subline of the EMT6 mouse mammary tumour. The culturing methods for log and early plateau phase monolayer cells and for large spheroids (800 $\mu$m in diameter) have been previously described (Kwok & Twentyman, 1985). The human SCLC cell line, POC, was kindly provided by Dr Morag Ellison, Ludwig Institute, Sutton, Surrey, UK. The maintenance methods used in this laboratory for SCLC lines have been described previously (Baillie-Johnson *et al.*, 1985). Cultures of POC were initiated by inoculating $10^6$ cells into 25 cm$^2$ flasks containing 5 ml medium or $5 \times 10^6$ cells into a 75 cm$^2$ flask containing 25 ml medium. The number of cells per flask increased progressively and cultures were used in log phase experiments after 5 days (at which time the 24 h labelling index after 1 $\mu$Ci ml$^{-1}$ $^3$HTdR is 1.0 (see **Results**)). In order to produce POC spheroids, aggregates, in 75 cm$^2$ flasks, were allowed to grow for 2 weeks after splitting. Thereafter, the aggregates were transferred to a stirrer bottle (Teche Ltd) containing 250 ml medium. The bottle was then gassed with a mixture of 5% $CO_2$ and 95% air for 3 min, sealed, and placed on a magnetic stirrer platform in a 37°C warm room. Medium change by replacing one half of the old medium with fresh medium was carried out once a week after the 2nd week of the spheroid culture. After 3 weeks, spheroids of 700 to 900 $\mu$m in size were selected for experiments.

*Culture medium*

The medium used for EMT6 mouse cells was Eagle's minimal essential medium with Earles's salts (Gibco BRL) supplemented with 20% new born calf serum (Gibco BRL). For human SCLC cells, the medium for cell culture and drug exposure experiments was RPMI 1640 medium (Gibco BRL) supplemented with 10% foetal calf serum (Gibco BRL). In clonogenic assay, the medium used was Ham's F12 medium (Gibco BRL) supplemented with 15% foetal calf serum.

*Experiments with EMT6 cells*

(*1*) *Cytotoxicity of* $^3$*HTdR* Graded doses of $^3$HTdR (specific activity = 53 Ci mmol$^{-1}$, Amersham International) firstly diluted in PBS were added into 5 ml culture medium in either 25 cm$^2$ flasks containing either day 2 (log phase) or day 5 (early plateau phase) monolayer cyltures (Twentyman *et al.*, 1975), or into 60 mm Petri dish base-coated with 1% Noble agar and containing 30 large spheroids ($\sim$800 $\mu$m in diameter). After 24 h incubation in a gassing incubator at 37°C, monolayer cultures or large spheroids, after rinsing twice with fresh medium, were trypsinized (with 5 ml 0.075% trypsin in PBS) into single cell suspensions. One half of the cells were then used for clonogenic assay whilst the remainder was used for autoradiography as described previously (Kwok & Twentyman, 1985).

(*2*) *Selective disaggregation of* $^3$*HTdR treated spheroids* Sixty large EMT6 spheroids were incubated in a 1% Noble agar base-coated 90 mm Petri dish containing 10 ml medium and 5 $\mu$Ci ml$^{-1}$ $^3$HTdR. After incubating for 24 h, spheroids were rinsed twice with fresh medium. Fifty spheroids then underwent selective disaggregation (in 5 ml 1 mg ml$^{-1}$ neutral protease (Type IX, Sigma Chemicals Co.)) as described previously (Kwok & Twentyman, 1985). The remaining 10 spheroids were fully disaggregated to produce a cell suspension designated 'Total'. After selective disaggregation, one half of the cells obtained from different fractions of spheroids were used for clonogenic assay whilst the other half was used for autoradiography.

(*3*) *Drug response of early plateau phase cells* (*with or without* $^3$*HTdR pretreatment*) In each experiment, a number of flasks containing cells in early plateau phase were firstly incubated with 5 $\mu$Ci ml$^{-1}$ $^3$HTdR in 5 ml medium whilst a number of similar flasks received no pretreatment. After 24 h incubation, the flasks were rinsed twice with 5 ml fresh medium. Five ml medium containing the appropriate concentrations of cytotoxic drugs adriamycin (ADM, Pharmitalia Ltd.), nitrogen mustard (HN2, Boots Co.) and CCNU (United States National Cancer Institute) were then added to pairs of flasks (i.e. with or without $^3$HTdR pretreatment). After 1 h incubation, cells were rinsed twice with 5 ml fresh medium. Single cell suspensions were obtained by trypsinization and clonogenic assay was then carried out.

(*4*) *Drug response of cells within large* (*800* $\mu$*m*) *spheroids, with or without* $^3$*HTdR pretreatment* Ninety large spheroids, in 15 ml medium with or without 5 $\mu$Ci ml$^{-1}$ $^3$HTdR, were incubated in a 1% Noble agar base-coated 90 mm Petri dish. After 24 h incubation, spheroids were rinsed twice with medium. Groups of 10 of the spheroids were then transferred into plastic universal tubes containing 5 ml medium. Thereafter, 5 ml medium containing 2 × the desired drug concentration were added into each corresponding pair of tubes, i.e. spheroids with or without $^3$HTdR pretreatment. After 1 h drug exposure, spheroids were rinsed twice with fresh medium, disaggregated into single cells and cell survival was then measured.

*Experiments with POC cells*

(*1*) *Cytotoxicity of* $^3$*HTdR towards POC cells* Five ml medium containing either 30 large POC spheroids, or cells in log phase (day 5) were incubated with graded doses of $^3$HTdR for 24 h. After incubation, cells or spheroids were rinsed twice with medium. Single cell suspensions were prepared by incubating the cells or spheroids in 3 ml 0.4% trypsin and 0.02% EDTA in PBS for 20 min at 37°C. Thereafter cells underwent both modified clonogenic assay and autoradiography.

(*2*) *Modified clonogenic assay for POC cells* The method used was based on the method described by Courtenay and Mills (1978) and has been previously described by Walls and Twentyman (1985). The main modification of the method was that the total volume of the agar plug was increased from 1 ml to 5 ml, i.e. 2 ml of cell suspension at 0.5 times the appropriate final dilution, 0.5 ml diluted August rat red blood cells and 2.5 ml of 0.6% Noble agar solution were added into a plastic test tube. The rest of the assay would then follow the same procedure as described by Walls and Twentyman (1985).

(*3*) *Drug response of cells from disaggregated POC spheroids* (*with or without* $^3$*HTdR pretreatment*) Ninety large POC spheroids (800 $\mu$m diameter) were incubated in 15 ml medium with or without 1 $\mu$Ci ml$^{-1}$ $^3$HTdR. After 24 h incubation, spheroids were rinsed twice with fresh medium. Seventy spheroids from each group were then disaggregated into single cells and resuspended in 35 ml. Five ml aliquots were transferred to plastic universal tubes and 5 ml medium containing twice the final concentration of drugs, ADM, HN2, CCNU and VCR (vincristine, Eli Lilly Ltd.) were added into each corresponding pair of tubes, i.e. with or without $^3$HTdR pretreatment. After 1 h incubation, cells were rinsed twice with fresh medium and underwent the modified clonogenic assay.

(*4*) *Drug response of cells from intact POC spheroids* (*with or without* $^3$*HTdR pretreatment*) The protocol was the same as that described for isolated spheroid cells, except that spheroids were disaggregated into a single cell suspension after cytotoxic drug exposure rather than immediately after 24 h incubation with $^3$HTdR and before cytotoxic drug exposure.

(*5*) *Response of log phase POC cells to cytotoxic drugs* A number of 75 cm$^2$ flasks of log phase POC cells (day 5) in 25 ml medium were firstly pooled and aggregates reduced to single cells by trypsinization. Ten ml of diluted single cell suspension were then transferred to plastic universal tubes and the appropriate amounts of cytotoxic drugs were added. After 1 h incubation, cells were rinsed twice with fresh medium and resuspended in 1 ml of medium. Cells then underwent the modified clonogenic assay.

### Results

*EMT6 cells*

(*1*) *Cytotoxicity of* $^3$*HTdR on EMT6 cells* Responses of EMT6 cells in log or early plateau phase monolayer growth or in large spheroids to $^3$HTdR alone are shown in Figure 1. In addition to surviving fraction, 1-LI of cells in early

**Figure 1** Response of EMT6 cells in (a) log phase, (b) early plateau phase and (c) large (800 μm) spheroids to $^3$HTdR. Surviving fraction (○——○), 1-labelling index (1-LI) (△---△). The s.d. of the LI at each dose of $^3$HTdR is < 10% of the mean value. In all figures, data from 2 independent experiments are shown.

plateau phase or within large spheroids is also included (where LI = the proportion of cells labelled with $^3$HTdR as determined by autoradiography). The dose response surve of log phase cells to $^3$HTdR (Figure 1a), is biphasic with an inflexion point at about 5 μCi ml$^{-1}$. The labelling index (LI) of log phase cells treated with more than 1 μCi ml$^{-1}$ $^3$HTdR is 1.0. For early plateau phase cells, the curves, for both surviving fraction and 1-LI after graded doses of $^3$HTdR, are coincident as are the two curves for cells within large spheroids (Figure 1b, c).

(*2*) *Selective disaggregation of $^3$HTdR treated large spheroids* Curves for the changes in surviving fraction or 1-LI of cells at different depths into large EMT6 spheroids after 5 μCi ml$^{-1}$ $^3$HTdR treatment are shown in Figure 2. The method for plotting this kind of curve has been previously described (Kwok & Twentyman, 1985). Basically, low values of % removed correspond to cells in the outer layers of the spheroids. Increasing values of % removed correspond to increasing depth. The surviving fraction (or 1-LI) of cells in the outer region of spheroids is lower than that of cells close to the centre and the shapes of the curves (for surviving fraction and for 1-LI) are similar.

**Figure 2** Response of cells in different regions of large (800 μm) EMT6 spheroids to 5 μCi ml$^{-1}$ $^3$HTdR. Surviving fraction (●, ○); 1-labelling index (1-LI) (▲, △). Open and closed symbols represent different experiments. The s.d. of the LI at different regions is < 10% of the mean value. Points are plotted at the midpoints of the % of the total cells isolated in the respective spheroid fraction (Kwok & Twentyman, 1985).

(*3*) *Drug response of early plateau phase EMT6 cells, with or without $^3$HTdR pretreatment* The responses of early plateau phase cells, with or without $^3$HTdR pretreatment, to graded doses of ADM, HN2 and CCNU are illustrated in Figure 3. The cell survival curves for $^3$HTdR pretreated cells have been normalized for the killing by $^3$HTdR alone. It may be seen that the response to ADM (Figure 3a) or HN2 (Figure 3c) of early plateau phase cells pretreated with $^3$HTdR is slightly lower than that of unpretreated cells. The sensitivity to CCNU is, however, unchanged by pretreatment (Figure 3b).

**Figure 3** Response of early plateau phase EMT6 cells to (a) ADM, (b) CCNU and (c) HN2. □———□: Cells pretreated with 5 μCi ml$^{-1}$ $^3$HTdR for 24 h. ○——○: Cells without pretreatment.

**Figure 4** Response of cells in large (800 μm) EMT6 spheroids to (a) ADM, (b) CCNU and (c) HN2. ○———○: Total cells. □——□: Cells in the inner region of spheroids. △——△: Cells in the outer region of spheroids (by calculation).

(*4*) *Response of cells in large spheroids to cytotoxic drugs* The responses to cytotoxic drugs of cells in large spheroids, either with or without $^3$HTdR pretreatment, are shown in Figure 4. The curve for cells in spheroids without $^3$HTdR pretreatment represent the response of the total cell population whilst the curves for cells from spheroids undergoing $^3$HTdR pretreatment are normalized for the killing by $^3$HTdR alone and hence represent the response of the cells surviving pretreatment. The response to cytotoxic drugs of those cells in spheroids killed by $^3$HTdR pretreatment can be calculated using equation (1):

$$SF_T = SF_0(1-a) + SF_i \times a \qquad (1)$$

where $SF_T$ : Surviving fraction of total spheroid cells.
$SF_0$ : Surviving fraction of spheroid cells killed by $^3$HTdR pretreatment ('labelled cells')
$SF_i$ : Surviving fraction of spheroid cells surviving $^3$HTdR pretreatment ('unlabelled cells')
$a$ : Surviving fraction of spheroid cells after $^3$HTdR exposure alone.

This equation has been used (together with values of $SF_T$ and $SF_i$ obtained in individual experiments at each drug dose) to deduce values of $SF_0$. These calculated values are also shown in Figure 4. The sensitivity of cells in spheroids to ADM (Figure 4a) and HN2 (Figure 4c) is therefore in the order, labelled cells > total cells > unlabelled cells, and to CCNU (Figure 4b) the order is unlabelled cells > total cells > labelled cells.

*POC cells*

(*1*) *Modified clonogenic assay* A very preliminary study on cell survival of logphase POC cells after 1 μCi ml$^{-1}$ $^3$HTdR treatment showed that, using the conventional 1 ml assay volume, the measured surviving fraction of cells plated at low number ($10^3$) was almost twice as high as the measured surviving fraction obtained when a higher number of cells ($10^4$) was plated. The differential is thought to be related to an 'indirect kill' effect by which cells which have not incorporated $^3$HTdR receive sufficient radiation dose from $^3$HTdR initially incorporated by other cells that they in turn are rendered non-clonogenic. A modified clonogenic assay using 5 ml assay volume rather than 1 ml is therefore used in order to dilute the effect by increasing the volume of assay medium. Results, as shown in Table I, indicate that even when the dose of $^3$HTdR is as high as 5 μCi ml$^{-1}$, the surviving fractions obtained from different dilutions of the same cells are essentially identical. Increase of assay volume from 1 ml to 5 ml does not however cause any change in the plating efficiency of POC cells (Table II). Therefore, throughout the whole study on POC cells, the modified clonogenic assay was used.

**Table I** 'Indirect Kill' effect on measured surviving fraction of $^3$HTdR treated POC cells

| *Volume of assay medium* | *$^3$HTdR dose* | *Measured surviving fraction* $10^3$ *cells plated* | *$10^4$ cells plated* |
|---|---|---|---|
| 1 ml | 1 μCi ml$^{-1}$ | 0.028 (16, 18)[a] | 0.015 (89, 100) |
| | 2.5 μCi ml$^{-1}$ | 0.0091 (8, 4) | 0 |
| 5 ml | 1 μCi ml$^{-1}$ | 0.13 (73, 63) | UC |
| | 5 μCi ml$^{-1}$ | 0.089 (48, 48) | 0.095 (487, 549) |

UC: uncountable (too many).
[a]Number in brackets is the actual number of colonies per assay tube.

**Table II** Plating efficiency of POC cells

| *Assay volume* | *Plating efficiency (%)* *Log phase* | *Spheroids* |
|---|---|---|
| 1 ml | 65 ± 3 | 47 ± 8 |
| 5 ml | 57 ± 4 | 47 ± 11 |

Figures are mean values (± s.d.) from 3 experiments.

(*2*) *Cytotoxicity of $^3$HTdR on POC cells* The responses of log phase POC cells and cells in large spheroids to graded doses of $^3$HTdR are shown in Figure 5. The dose response curve for log phase cells to $^3$HTdR is biphasic. The LI of these cells is ~0.9 at a dose of 0.5 μCi ml$^{-1}$ and almost 1.0 when treated with more than 1 μCi ml$^{-1}$. The curves for both surviving fraction and 1-LI of cells in large spheroids after 24 h incubation with more than 1 μCi ml$^{-1}$ $^3$HTdR are parallel to the abscissa. However, the surviving fraction is always slightly higher than 1-LI at each tested $^3$HTdR dose.

(*3*) *Drug response of cells from disaggregated spheroids, with or without $^3$HTdR pretreatment* Response curves for cells disaggregated from large spheroids immediately before exposure to ADM, CCNU, HN2 or VCR (i.e. isolated spheroid cells) are shown in Figure 6. The surviving fractions of isolated cells from spheroids receiving $^3$HTdR pretreatment have been normalized for killing by $^3$HTdR alone. The responses of isolated cells from unpretreated spheroids to ADM (Figure 6a), CCNU (Figure 6b), HN2 (Figure 6c) and VCR (Figure 6d) are all similar to those of the cells which survive $^3$HTdR pretreatment.

(*4*) *Drug response of cells in large intact spheroids, with or without $^3$HTdR pretreatment* The responses of cells in large intact POC spheroids, either with or without $^3$HTdR pretreatment, to different cytotoxic drugs are shown in Figure 7. There are 3 curves in each figure, i.e. curves for total, labelled and unlabelled cells. The curve for unlabelled cells

Figure 5 Response of POC cells in log phase (□) and large (800 μm) spheroids (△, ▲) to $^3$HTdR. Open symbols: surviving fraction. Closed symbols: 1-LI. The s.d. of the LI at each dose of $^3$HTdR is < 10% of the mean value.

Figure 6 Response of POC isolated spheroid cells to (a) ADM, (b) CCNU, (c) HN2 and (d) VCR. □– – – –□: Cells disaggregated from intact spheroids pretreated with 1 μCi ml$^{-1}$ $^3$HTdR for 24 h. ○——○: Cells disaggregated from untreated spheroids.

Figure 7 Response of cells in large (800 μm) intact POC spheroids to (a) ADM, (b) CCNU, (c) HN2 and (d) VCR. ○: Total cells in large spheroids. □: Cells in the inner region of spheroids. △: Cells in the outer region of spheroids (by calculation).

(i.e. cells surviving in spheroids receiving $^3$HTdR treatment) has already been normalized for cell kill by 1 μCi ml$^{-1}$ $^3$HTdR alone. The curve for labelled cells in spheroids is plotted in accordance with the equation (1). The sensitivity of cells in spheroids to VCR and ADM is in the order labelled cells > total cells > unlabelled cells; to CCNU the order is unlabelled cells > total cells > labelled cells, whilst to HN2 it is labelled cells = total cells = unlabelled cells.

(5) *Response of log phase POC cells to cytotoxic drugs* The responses of log phase POC cells to cytotoxic drugs are shown in Figure 8. In addition, response curves for isolated spheroid cells, without $^3$HTdR pretreatment, are adopted from Figure 6. Compared with isolated spheroid cells, log phase cells are more sensitive to ADM and VCR but have similar sensitivity to CCNU and HN2.

### Discussion

### *EMT6 cells*

There are three questions which have to be answered before the $^3$HTdR suicide method can be applied in the study of the influence of tumour geometry on response of cells in spheroids to cytotoxic drugs. Firstly, are the cells killed by $^3$HTdR those cells which have incorporated $^3$HTdR into their DNA during synthesis? Secondly, is it true that cells in the outer region of spheroids are more likely to be killed by high dose $^3$HTdR than are cells near to the centre of spheroids? Thirdly, will cells which have survived $^3$HTdR

**Figure 8** Response of POC cells to (a) ADM, (b) CCNU, (c) HN2 and (d) VCR. Isolated spheroid cells (○———○); Log phase cells (△——△).

pretreatment respond differently to cytotoxic drugs as compared to cells without $^3$HTdR pretreatment?

(*1*) *Surviving fraction* versus *1-LI* To answer the first question raised, clonogenic cell survival assay and autoradiographic determination of LI were used concomitantly to study the response to $^3$HTdR of EMT6 cells in either log or early plateau phase or in large spheroids. The good agreement seen between these 2 parameters strongly supports the idea that the cells killed by $^3$HTdR treatment are the labelled cells. The LI of early plateau cells after 24 h incubation with $^3$HTdR is ~0.45 which agrees with the result obtained for another EMT6 subline - EMT6/CC (Twentyman *et al.*, 1975). For log phase cells, as shown in Figure 1a, the cell survival curve is biphasic with an inflexion point at about 5 μCi ml$^{-1}$. The LI at all doses of $^3$HTdR tested is essentially 1.0. The differential between changes of surviving fraction and 1-LI for dose below 5 μCi ml$^{-1}$ may indicate that at low doses, the amount of $^3$HTdR incorporated by log phase cells may not be high enough to kill all the labelled cells. For plateau and spheroid cells, however, (Figure 1b, c), the lack of a dose-response relationship indicates that a dose of 1 μCi ml$^{-1}$ is sufficient to kill all susceptible cells. A dose of 5 μCi ml$^{-1}$ of $^3$HTdR (the minimal dose which can kill essentially all the cycling cells within the various populations) was therefore chosen for future experiments.

(*2*) *Regional specificity of $^3$HTdR cell kill in spheroids* The autoradiography of large EMT6 spheroids after 24 h incubation with 1 μCi ml$^{-1}$ $^3$HTdR has shown that $^3$HTdR labelled cells are mainly located in the outer 50 μm rim of spheroids while there are relatively few labelled cells in the deeper region of the spheroids (Kwok, 1986). In an attempt to examine the correlation between the patterns for $^3$HTdR labelling and $^3$HTdR cell kill of cells in spheroids, using the selective disaggregation method, surviving fraction and 1-LI of cells at different depths in spheroids were measured. Both parameters were found to be much lower for cells in the outer region of spheroids than for cells near to the centre. The two curves, surviving fraction and 1-LI, are similar in shape to each other but the value of surviving fraction is always higher than 1-LI. The trend for surviving fraction along the radius of spheroids indicates that cells in the outer region of spheroids are more likely to be killed by $^3$HTdR treatment than cells nearer the centre. The similarity between the curves for surviving fraction and 1-LI confirms that cell kill by $^3$HTdR is closely related to cellular $^3$HTdR incorporation. The higher value of surviving fraction compared with 1-LI implies that some of the cells have incorporated a sublethal amount of $^3$HTdR and can therefore still survive. Although 5 μCi ml$^{-1}$ $^3$HTdR is a sufficiently high dose to give a good agreement between surviving fraction and 1-LI for log phase EMT6 cells, it may not be high enough for some cells in spheroids where the cell kinetic parameters, such as cell cycle time, may be quite different to those for log phase cells (Kwok & Twentyman, 1985). Although, therefore, in this discussion we will use the term 'cycling cells' to indicate cells in spheroids killed by the $^3$HTdR suicide technique, it should be borne in mind that a small proportion of the cycling cells may, in fact, escape such killing.

(*3*) *Influence of $^3$HTdR pretreatment on response of cells to cytotoxic drugs* In an attempt to find out the influence of $^3$HTdR pretreatment on the drug response of cells surviving such treatment, EMT6 cells in early plateau phase are used instead of large intact spheroids. These populations have a similar cell survival after $^3$HTdR treatment and factors related to spheroid structure, such as penetration of drugs, would complicate the interpretation of the results in the spheroid system.

Early plateau phase cells with $^3$HTdR pretreatment are a little less sensitive to ADM than are untreated cells (Kwok & Twentyman, 1985). This is the opposite result than that expected if the pretreatment were producing sublethal damage in potentially surviving cells which was able to interact with ADM damage. The result obtained probably reflects selective killing of the actively cycling (and hence ADM sensitive) proportion of the plateau phase population (Kwok & Twentyman, 1985). A similar explanation will also account for the small differential sensitivity seen for HN2 (Figure 3c). For CCNU, pretreatment has no effect on the response of those cells which survive. It may therefore be concluded that use of the $^3$HTdR suicide technique is valid in that it does not sensitise cells which survive the procedure to subsequent cytotoxic drug treatment.

(*4*) *$^3$HTdR suicide method on large EMT6 spheroids* With satisfactory answers to the above three questions, experiments using the $^3$HTdR suicide technique to study responses of cells in different regions of large EMT6 spheroids to cytotoxic drugs were therefore carried out. Since a high dose of $^3$HTdR kills cycling rather than non-cycling cells (Becker *et al.*, 1965; Rockwell *et al.*, 1976) and cycling cells within spheroids are mainly located in the outer rim while non-cycling cells are mainly near to the centre, results for cells surviving $^3$HTdR pretreatment may be taken as essentially measuring the sensitivity of inner cells. Cells in the outer region were found to be more sensitive to ADM and to HN2 than are cells near the centre whilst the opposite pattern was found for CCNU. These results are in good agreement with those obtained using the selective disaggregation method (Kwok & Twentyman, 1985), and use of the $^3$HTdR suicide method is therefore further validated for subsequent use in the POC system.

*POC cells*

(*1*) *Log phase cells* versus *isolated spheroid cells* Cell cycle distribution is one of the factors governing response of cells in spheroids to cytotoxic drugs (Kwok & Twentyman, 1985). To separate this factor from the other factors, a study of the response to cytotoxic drugs of POC cells in log phase versus isolated spheroid cells was carried out and the result is shown in Figure 8. Isolated spheroid cells are cells from large spheroids ($\sim 800\,\mu$m in diameter) which are prepared by disaggregation of large spheroids just before drug exposure. For log phase cells, the LI following 24 h exposure to $1\,\mu$Ci ml$^{-1}$ $^3$HTdR is 1.0 whilst for cells in large spheroids or isolated spheroid cells it is around 0.6 (Figure 5). POC cells in log phase, as shown in Figure 8, are more sensitive to ADM and VCR than are isolated spheroid cells. This differential sensitivity to these 2 drugs (cycling cells more sensitive) is as previously reported in other systems (Wibe *et al.*, 1981; Kwok & Twentyman, 1985). The responses of log phase cells to CCNU or HN2 are similar to those of isolated spheroid cells. Although in the EMT6 system plateau phase cells were more sensitive to CCNU than log phase cells (Kwok & Twentyman, 1985), the differential was only small and the target populations (in terms of LI) were more different from each other than those used in the present POC study. The cytotoxicity of HN2 on POC cells is similar in both log phase and isolated spheroid cells. In EMT6 cells, there was a greater sensitivity to HN2 in log phase compared to early plateau phase (Kwok & Twentyman, 1985) whereas the opposite has been reported for V79 cells (Hetzel & Kaufman, 1983). With regard to proliferation dependence, cell kill by HN2 is therefore cell line dependent.

(*2*) *$^3$HTdR suicide method* Results from EMT6 cells have shown that the $^3$HTdR suicide method can be validly used to study the relative sensitivity to cytotoxic drugs of cells in the outer and inner regions of spheroids. To apply this technique in the POC system, however, the three questions mentioned in the section for EMT6 cells have again to be considered.

*2.1 Surviving fraction* versus *labelling index* In Figure 5, the responses of log phase POC cells and cells in large POC spheroids to graded doses of $^3$HTdR are shown. The curves are very similar. The basic similarity between the curves would indicate that the cells killed are those incorporating the most $^3$HTdR. For both log phase cells and spheroid cells, there is little further cell kill above a $^3$HTdR dose of $1\,\mu$Ci ml$^{-1}$ indicating that this dose is sufficiently high to kill essentially all susceptible cells. We therefore used a dose of $1\,\mu$Ci ml$^{-1}$ in subsequent experiments, not wishing to go to higher doses because of the possibility of the 'indirect killing' effect previously mentioned. For log phase cells, the labelling index of cells on autoradiographs was 1.0 at this dose of $^3$HTdR, compared with a surviving fraction of $\sim 10\%$. Similarly, the points for surviving fraction of spheroid cells lie a little above the points for 1-LI. It must again therefore be borne in mind that a small proportion of cycling cells may escape killing by $^3$HTdR in the suicide technique used here.

*2.2 Geometric specificity of $^3$HTdR cell kill in POC spheroids* After 24 h incubation with $1\,\mu$Ci ml$^{-1}$ $^3$HTdR, labelled cells are found mainly in the outer rather than the inner regions of spheroids (Kwok, 1986). Thus, it is likely that cell kill by $^3$HTdR incorporation will mainly occur in the outer region rather than in the inner region of spheroids. Although correspondence between cell kill and $^3$HTdR incorporation at different depths in POC spheroids cannot be directly proved, evidence from the EMT6 system mentioned above strengthens the agreement provided by the autoradiographic study.

*2.3 Influence of $^3$HTdR pretreatment on response of POC cells to cytotoxic drugs* In an attempt to investigate the influence of $^3$HTdR pretreatment on the response of POC cells to cytotoxic drugs, experiments comparing the response to drugs of isolated spheroid cells, with or without $^3$HTdR pretreatment, were carried out and results are shown in Figure 6. In this set of experiments, isolated cells are preferred because of the complication of spheroid structure related factors involved if POC aggregates from any growth state are used. Isolated spheroid cells with $^3$HTdR pretreatment are obtained by disaggregation, just before drug exposure, of large intact spheroids which have been preincubated with $1\,\mu$Ci ml$^{-1}$ $^3$HTdR for 24 h.

The sensitivity of isolated spheroid cells to ADM, CCNU, HN2 and VCR (Figure 6) are essentially unchanged by $^3$HTdR pretreatment. It may have been expected that killing of the cycling cells would leave a population relatively resistant to ADM and VCR (as found for ADM in EMT6 in the analogous experiments). However, the different sensitivity to ADM between log phase POC cells and POC cells isolated from large spheroids is considerably less than that between log and early plateau phase EMT6 cells. Where the differential sensitivity for POC is greater (i.e. for VCR), a small reduction in sensitivity following $^3$HTdR pretreatment cannot be ruled out. The general indication of the data is that $^3$HTdR pretreatment has no major influence on the cytotoxic drug sensitivity of the surviving cells.

*2.4 Differential response of cells in large POC spheroids to cytotoxic drugs* On the basis of the above studies, the $^3$HTdR suicide technique was used in large POC spheroids and results for the response of cells in the outer and inner regions of spheroids to 4 cytotoxic drugs, ADM, VCR, CCNU and HN2, are summarized in Figure 7. In addition to the inherent cellular response characters, drug response of cells in spheroids is governed by 4 factors, cell cycle distribution, intercellular contact effect, microenvironment and drug penetrability. The latter three have been collectively termed 'spheroid structural factors' (Kwok & Twentyman, 1985). In this paper, only 3 of the 4 factors, microenvironment, drug penetration and cell cycle distribution, modulating response of cells in spheroids to cytotoxic drugs will be considered. The remaining factor, i.e. the intercellular contact effect, has been shown to have little or no influence on the response of POC cells to the 4 cytotoxic drugs studied (Kwok, 1986).

Cells in the outer region of spheroids are found to be more sensitive to ADM than are cells in the inner region (Figure 7a). As cycling cells are more sensitive to ADM than are non-cycling cells (Figure 8a), this differential may partly be due to the difference of cell cycle distribution throughout the spheroids, i.e. more cycling cells are found in the outer region whilst the inner region of spheroids is mostly composed of non-cycling cells (Kwok & Twentyman, 1985). Microfluorographs of POC spheroids exposed to $15\,\mu$g ml$^{-1}$ ADM for 3 h (Kwok, 1986) has demonstrated a limited penetrability of ADM in these spheroids and this may imply that the response differential amongst cells in spheroids to ADM may also be due to the limited penetrability of ADM in spheroids. Amongst the three factors possibly governing the response of cells in spheroids to cytotoxic drugs, as in the EMT6 system (Kwok & Twentyman, 1985), the factors of cell cycle distribution and penetrability of ADM have been shown to play a major role and appear to be sufficient to explain fully the response pattern of cells in POC spheroids to ADM. It is not possible from the data to ascertain whether changes in microenvironment play any additional role.

Cells in the outer region of spheroids are also more sensitive to VCR than are cells in the inner area. Such differential, as the case for ADM, can also be explained partly on the basis of cell cycle distribution throughout the spheroids. If the response to VCR of cells in intact spheroids, either with or without $^3$HTdR pretreatment (Figure 7d) is compared with the response of isolated spheroid cells after the same pretreatment (Figure 6d), cells in intact spheroids are seen to be more sensitive. In addition to the factor of cell cycle distribution which will be similar in the above comparison, other spheroid structure related factors may therefore also be involved. It is unlikely to be an intercellular contact effect as the influence of this factor on the response to VCR of POC cells, compared with the differential found in the above comparison, is too small (Kwok, 1986). Therefore, the two remaining factors, limited drug penetration (which has been demonstrated in another spheroid system (Wibe & Oftebro, 1981), and microenvironment may possibly also be important in determining the response of cells in POC spheroids to VCR.

In contrast to the results for VCR and ADM, the sensitivity of cells in spheroids to CCNU is greater in the inner compared with the outer region (Figure 7b). The factor of cell cycle distribution should have no influence on such differential as cycling and non-cycling cells respond similarly to CCNU (Figure 8b). Studies on large EMT6 spheroids, have demonstrated that $^{14}$C-CCNU becomes distributed evenly throughout the spheroid (Kwok & Twentyman, 1985). It is therefore likely that the distribution of CCNU in POC spheroids will also be uniform. Cells treated in intact POC spheroids (either total cells, or cells in the outer or inner regions), are always far more sensitive to CCNU than are corresponding cells treated as isolated spheroid cells. Therefore, amongst the 4 factors which may influence the response of cells in spheroids to CCNU, the microenvironmental factor appears to predominate.

The pattern of response of cells within POC spheroids to HN2 is quite different to that to the other three drugs. Regardless of their location within spheroids, cells respond to HN2 similarly (Figure 7c) and thus tumour geometry is not important in determining the response of POC cells to HN2. This result is different to the finding from EMT6 spheroids where cells in the outer region were more sensitive to HN2 than cells in the inner region. It was concluded, however, that the response differential in the EMT6 system was a result of the differential sensitivity to HN2 of cycling versus non-cycling EMT6 cells (Kwok & Twentyman, 1985). As this differential does not exist for POC cells (Figure 8c), the finding in POC spheroids concurs with the conclusion that cell cycle distribution is the major factor determining response to HN2 of cells at different depths in spheroids.

### References

BAILLIE-JOHNSON, H., TWENTYMAN, P.R. FOX, N.E. & 6 others (1985). Establishment and characterization of cell lines from patients with lung cancer (predominantly small cell carcinoma). *Br. J. Cancer*, **52**, 495.

BECKER, A.J., McCULLOCH, E.A., SIMINOVITCH, L. & TILL, J.E. (1965). The effect of different demands for blood cell production on DNA synthesis by hemopoietic colony-forming cells of mice. *Blood*, **26**, 296.

COURTENAY, V.D. & MILLS, J. (1978). An *in vitro* colony assay for human tumours grown in immune-suppressed mice treated *in vivo* with cytotoxic drugs. *Br. J. Cancer*, **37**, 261.

DURAND, R.E. (1982). Use of Hoechst 33342 for cells selection from multicell systems. *J. Histochem. Cytochem.*, **30**, 117.

FREYER, J.P. & SUTHERLAND, R.M. (1980). Selective dissociation and characterization of cells from different regions of multicell tumour spheroids. *Cancer Res.*, **40**, 3956.

HETZEL, F.W. & KAUFMAN, N. (1983). Chemotherapeutic drugs as indirect oxygen radiosensitizers. *Int. J. Radiat. Oncology Biol. Phys.*, **9**, 751.

KWOK, T.T. (1986). The influence of tumour geometry upon cellular response to cytotoxic agents: An *in vitro* study using multicellular spheroids. *Ph.D. Thesis, University of Cambridge.*

KWOK, T.T. & TWENTYMAN, P.R. (1985). The relationship between tumour geometry and the response of tumour cells to cytotoxic drugs – an *in vitro* study using EMT6 multicellular spheroids. *Int. J. Cancer* **35**, 675.

ROCKWELL, S., FRINDEL, E. & TUBIANA, M. (1976). A technique for determining the proportion of the clonogenic cells in S phase in EMT6 cell cultures and tumours. *Cell Tissue Kinet.*, **9**, 313.

TWENTYMAN, P.R., WATSON, J.V., BLEEHEN, N.M. & ROWLES, P.M. (1975). Changes in cell proliferation kinetics occuring during the life history of monolayer cultures of a mouse tumour cell line. *Cell Tissue Kinet.*, **8**, 41.

WALLS, G.A. & TWENTYMAN, P.R. (1985). Cloning of human lung cancer cells. *Br. J. Cancer*, **52**, 505.

WIBE, E. & OFTEBRO, R. (1981). A study of factors related to the action of 1-proparyl-5-chloropyrimidine-2-one (NY3170) and vincristine in human multicellular spheroids. *Int. J. Cancer Clin. Oncol.*, **17**, 1053.

WU, A.M. (1981). A method for measuring the generation time and length of DNA synthesizing phase of clonogenic cells in a heterogenous population. *Cell Tissue Kinet.*, **14**, 39.